# Supplementary material for: The Effect of Previous Exposure to Malaria Infection and Clinical Malaria Episodes on the Immune Response to the Two-Dose Ad26.ZEBOV, MVA-BN-Filo Ebola Vaccine Regimen
Source: Vaccines (Basel). 2023 Aug 2;11(8):1317. doi: 10.3390/vaccines11081317 (PMC10459393; doi:10.3390/vaccines11081317)
Supplement: Supplementary file 1 [file vaccines-11-01317-s001.zip › vaccines-2511339-supplementary.pdf]

The Effect of Previous Exposure to Malaria Infection and Clinical Malaria Episodes on the Immune Response to the Two-Dose Ad26.ZEBOV, MVA-BN-Filo Ebola Vaccine Regimen

Supplementary Material

Table of Contents

1. SUPPLEMENTARY METHODS....2

1.1. Luminex xMAP Technique....2

1.2. Data Validation of the Categorical Variable of Previous Exposure to Malaria 2

2. SUPPLEMENTARY TABLES....3

Supplementary Table S1 3

Supplementary Table S2. 4

Supplementary Table S3. 5

Supplementary Table S4 6

Supplementary Table S5 7

Supplementary Table S6 8

Reference 8

1. SUPPLEMENTARY METHODS

1.1. Luminex xMAP Technique

The Luminex xMAP technique (Luminex Corp, Austin TX) offers the advantage of simultaneously detecting and quantifying antibodies to multiple antigens. Colour-coded beads are coated with malaria antigens (each bead colour is coated with a different antigen) before the sample (serum, plasma, etc.) is added. If the sample contains a specific antibody against a malaria antigen coating the beads, the antibody binds to the antigen and forms an antigen-antibody complex. A secondary fluorescent antibody is then added, which binds the fragment crystallizable region (Fc region) of the sample antibody in each antigen-antibody complex. A MAGPIX analyser aspirates and transports the beads into its imaging chamber, exposing them to LED lights. These excite the fluorescent molecule attached to the antibody-antigen complexes leading to the emission of fluorescent light whose intensity is directly proportionate to the amount of antibody-antigen binding. The machine detects the fluorescence and sorts the data for each antigen according to the colour code of the bead. The analyser counts every bead for each antigen per sample and takes a median value. The final reading is the Median Fluorescent Intensity (MFI).

1.2. Data Validation of the Categorical Variable of Previous Exposure to Malaria

In a previous study by Achan et al. [1], which employed the Luminex MAGPIX platform, antibody responses to the following six *Plasmodium falciparum* (*P. falciparum*) antigens were considered the most appropriate to determine exposure to malaria infection: apical membrane antigen 1 (AMA-1), merozoite surface protein 1.19 (MSP-1.19), and glutamate-rich protein (GLURP.R2) reflecting long-term exposure to malaria; reticulocyte-binding protein homologue (Rh2.2030), gametocyte exported protein (GEXP18), and early transcribed membrane protein (Etramp5.Ag1) reflecting recent exposure to malaria (i.e., infection in the past ~9 months). Antibody responses to each antigen are expressed as MFI (see above 1.1 Luminex xMAP technique).

To check if the categories of previous exposure to malaria, which were obtained by combining the MFI to the six *P. falciparum* antigens, adequately summarised the MFI of each antigen, we evaluated how the MFI for each antigen varied according to the categories of previous exposure to malaria.

The mean and the median MFI for each *P. falciparum* antigen increased consistently with increasing age-adjusted categories of previous exposure to malaria, being lower in the low-exposure group, intermediate in the intermediate-exposure group and higher in the high-exposure group (Supplementary Table S6). This showed that the previous exposure to malaria categorisation was able to adequately summarise the immune response to each of the six *P. falciparum* antigens, as expected.

2. SUPPLEMENTARY TABLES

Supplementary Table S1. Sociodemographic characteristics of the malaria study participants.

Characteristic		n (%) N=587
Age cohort	1-3 years	125 (21)
	4-11 years	133 (23)
	12-17 years	141 (24)
	≥ 18 years	188 (32)
Sex	Male	368 (63)
	Female	219 (37)
Ethnicity	Themne	405 (69)
	Limba	71 (12)
	Soso	50 (9)
	Mende	24 (4)
	Fula	14 (2)
	Other ethnicities	23 (4)
Religion	Muslim	484 (82)
	Christian	100 (17)
	None or not stated	3 (1)
Level of Education	No formal education	160 (27)
	Primary (1-6 grades)	200 (34)
	Secondary/High School	213 (36)
	Tertiary level	14 (2)

Supplementary Table S2. Ebola Virus (EBOV) Glycoprotein (GP)-specific binding antibody geometric mean concentrations (GMCs) post dose 1 (measured on Day 57) by categories of previous exposure to malaria, based on participants' serologic response to a panel of *Plasmodium falciparum (P. falciparum)* long-term exposure antigens [1] at the screening visit, overall and by age cohort.

Long-term Exposure to Malaria at Screening	N (%)	Post-Dose 1 EBOV GP-Specific Binding Antibody GMC, EU/mL	GMR [2] (95% CI)	*p*
All participants [3]	N=474			
Low	177 (37.3)	344 (295-401)	1	0.39
Intermediate	180 (38.0)	325 (283-373)	0.93 (0.77-1.14)	
High	117 (24.7)	418 (347-504)	1.09 (0.86-1.37)	
By age group				
1-3 years	N=96			
Low	37 (38.5)	852 (646-1123)	1	0.51
Intermediate	28 (29.2)	655 (518-828)	0.79 (0.55-1.14)	
High	31 (32.3)	733 (516-1041)	0.87 (0.55-1.37)	
4-11 years	N=116			
Low	45 (38.8)	300 (228-395)	1	0.18
Intermediate	42 (36.2)	428 (334-547)	1.39 (0.96-2.00)	
High	29 (25.0)	452 (320-637)	1.13 (0.76-1.67)	
12-17 years	N=115			
Low	43 (37.4)	367 (279-482)	1	0.67
Intermediate	44 (38.3)	297 (226-390)	0.87 (0.60-1.25)	
High	28 (24.3)	318 (226-448)	0.89 (0.61-1.29)	
≥ 18 years	N=147			
Low	52 (35.4)	192 (149-249)	1	0.83
Intermediate	66 (44.9)	215 (172-269)	0.96 (0.72-1.27)	
High	29 (19.7)	276 (192-397)	1.06 (0.76-1.48)	

[1]Apical membrane antigen 1 (AMA-1), merozoite surface protein 1.19 (MSP-1.19) and glutamate-rich protein (GLURP.R2). [2]Adjusted for baseline EBOV GP-specific antibody concentrations. [3]Categories of previous exposure to malaria are age-adjusted. GMR=geometric mean ratio.

Supplementary Table S3. EBOV GP-specific binding antibody GMCs post-dose 2 (measured on Day 78) by categories of previous exposure to malaria, based on participants' serologic response to a panel of *P. falciparum* long-term exposure antigens [1] at the screening visit, overall and by age cohort.

**Long-Term Exposure to Malaria at Screening**	**N (%)**	**Post-Dose 2 EBOV GP-Specific Binding Antibody GMC, EU/mL**	**GMR [2] (95% CI)**	***p***
All participants [3]	N=466			
Low	176 (37.8)	7908 (6571- 9516)	1	0.50
Intermediate	174 (37.3)	8673 (7176-10482)	1.15 (0.88-1.49)	
High	116 (24.9)	9212 (7206-11777)	1.20 (0.88-1.63)	
By age group				
1-3 years	N=96			
Low	37 (38.5)	22596 (17121-29823)	1	0.98
Intermediate	28 (29.2)	22762 (16540-31324)	1.06 (0.66-1.70)	
High	31 (32.3)	21404 (11677-39233)	1.00 (0.56-1.77)	
4-11 years	N=115			
Low	45 (39.1)	9000 (6463-12532)	1	0.06
Intermediate	41 (35.7)	13835 (9935-19266)	1.64 (1.03-2.62)	
High	29 (25.2)	7601 (5100-11329)	0.95 (0.54-1.65)	
12-17 years	N=112			
Low	43 (38.4)	8848 (5960-13137)	1	0.49
Intermediate	42 (37.5)	11725 (8329-16506)	1.32 (0.78-2.23)	
High	27 (24.1)	9359 (6624-13223)	1.04 (0.62-1.74)	
≥ 18 years	N=143			
Low	51 (35.7)	2996 (2413-3720)	1	0.40
Intermediate	63 (44.0)	3409 (2623-4431)	1.15 (0.81-1.64)	
High	29 (20.3)	4468 (3105-6428)	1.34 (0.89-2.02)	

[1]Apical membrane antigen 1 (AMA-1), merozoite surface protein 1.19 (MSP-1.19) and glutamate-rich protein (GLURP.R2). [2]Adjusted for baseline EBOV GP-specific antibody concentrations. [3] Categories of previous exposure to malaria are age-adjusted.

Supplementary Table S4. EBOV GP-specific binding antibody GMCs post dose 1 (measured on Day 57) by categories of previous exposure to malaria, based on participants' serologic response to a panel of *P. falciparum* recent exposure antigens [1] at the screening visit, overall and by age cohort.

**Recent Exposure to Malaria at Screening**	**N (%)**	**Post-dose 1 EBOV GP-Specific Binding Antibody GMC, EU/mL**	**GMR [2] (95% CI)**	***p***
All participants [3]	N=474			
Low	170 (35.9)	364 (313-424)	1	0.41
Intermediate	187 (39.4)	335 (290-388)	0.88 (0.72-1.07)	
High	117 (24.7)	367 (307-438)	0.97 (0.78-1.22)	
By age group				
1-3 years	N=96			
Low	42 (43.8)	741 (584-940)	1	0.96
Intermediate	24 (25.0)	771 (573-1038)	0.96 (0.65-1.42)	
High	30 (31.2)	750 (521-1081)	0.95 (0.61-1.49)	
4-11 years	N=116			
Low	34 (29.3)	344 (243-487)	1	0.66
Intermediate	53 (45.7)	411 (331-511)	0.99 (0.66-1.48)	
High	29 (25.0)	361 (255-511)	0.84 (0.53-1.34)	
12-17 years	N=115			
Low	43 (37.4)	340 (255-452)	1	0.88
Intermediate	43 (37.4)	304 (227-408)	0.92 (0.64-1.32)	
High	29 (25.2)	344 (261-452)	0.94 (0.66-1.36)	
≥ 18 years	N=147			
Low	51 (34.7)	224 (176-286)	1	0.71
Intermediate	67 (45.6)	225 (173-293)	1.05 (0.78-1.42)	
High	29 (19.7)	190 (151-238)	0.92 (0.68-1.24)	

[1]Reticulocyte-binding protein homologue (Rh2.2030), gametocyte exported protein (GEXP18) and early transcribed membrane protein (Etramp5.Ag1). [2]Adjusted for baseline EBOV GP-specific antibody concentrations. [3] Categories of previous exposure to malaria are age-adjusted.

Supplementary Table S5. EBOV GP-specific binding antibody GMCs post-dose 2 (measured on Day 78) by categories of previous exposure to malaria, based on participants' serologic response to a panel of *P. falciparum* recent exposure antigens [1] at the screening visit, overall and by age cohort.

Recent Exposure to Malaria at Screening	N (%)	Post-dose 2 EBOV GP-Specific Binding Antibody GMC, EU/mL	GMR [2] (95% CI)	*p*
All participants [3]	N=466			
Low	169 (36.3)	9003 (7345- 11037)	1	0.91
Intermediate	181 (38.8)	8174 (6836-9774)	0.94 (0.71-1.23)	
High	116 (24.9)	8318 (6587-10502)	0.98 (0.73-1.33)	
By age group				
1-3 years	N=96			
Low	42 (43.8)	23257 (17613-30709)	1	0.86
Intermediate	24 (25.0)	25239 (17305-36810)	1.04 (0.62-1.76)	
High	30 (31.2)	18911 (10462-34184)	0.89 (0.50-1.58)	
4-11 years	N=115			
Low	34 (29.6)	12534 (8378-18750)	1	0.28
Intermediate	52 (45.2)	10321 (7740-13763)	0.87 (0.53-1.43)	
High	29 (25.2)	7405 (4932-11118)	0.64 (0.36-1.14)	
12-17 years	N=112			
Low	43 (38.4)	9216 (5804-14636)	1	0.50
Intermediate	40 (35.7)	11757 (9006-15348)	1.28 (0.74-2.19)	
High	29 (25.9)	8914 (6619-12005)	0.95 (0.55-1.64)	
≥ 18 years	N=143			
Low	50 (35.0)	3175 (2543-3964)	1	0.66
Intermediate	65 (45.4)	3577 (2710-4721)	1.12 (0.79-1.59)	
High	28 (19.6)	3622 (2705-4850)	1.20 (0.83-1.74)	

[1]Reticulocyte-binding protein homologue (Rh2.2030), gametocyte exported protein (GEXP18) and early transcribed membrane protein (Etramp5.Ag1). [2]Adjusted for baseline EBOV GP-specific antibody concentrations. [3] Categories of previous exposure to malaria are age-adjusted.

Supplementary Table S6. Serologic responses (Median Fluorescent Intensity) to *Plasmodium falciparum* antigens indicative of long-term and recent exposure to malaria infection, by category of previous exposure to malaria.

***Plasmodium falciparum* Antigens** [1]		**Serologic Responses (Median Fluorescent Intensity** [2]**)**		
		**Low-Exposure to Malaria** N=144	**Intermediate-Exposure to Malaria** N=213	**High-Exposure to Malaria** N=117
Long-term exposure				
AMA-1	mean (SD)	15654.6 (11143.0)	24064.1 (8768.3)	26863.4 (7682.4)
	median (IQR)	16314.5 (2845.5-25930.5)	27130.0 (22271.0- 29568.0)	29028.0 (25181.0- 31246.0)
MSP1.19	mean (SD)	6712.3 (7101.6)	17905.1 (9567.8)	23848.1 (8592.5)
	median (IQR)	4397.0 (1280.5-9883.3)	18811.0 (8932.5-26674.0)	26976.5 (18627.0-30274.0)
GLURP.R2	mean (SD)	13402.2 (14660.0)	27037.2 (15529.3)	30453.1 (14693.6)
	median (IQR)	5888.0 (452.3-27124.5)	33506.5 (10232.0-39559.5)	36470.0 (19701.0-41219.0)
Recent exposure				
Rh2.2030	mean (SD)	7327.7 (7693.1)	15210.4 (9646.5)	19892.2 (10622.2)
	median (IQR)	4249.0 (966.0-12496.8)	15339.0 (6800.0-23962.0)	22729.0 (10209.0-29284.0)
GEXP18	mean (SD)	1401.2 (1050.3)	3343.5 (2327.0)	5204.7 (3291.0)
	median (IQR)	1100.5 (614.0-1863.8)	2624.0 (1695.0-4490.0)	4895.5 (2708.0-6875.5)
Etramp5.Ag1	mean (SD)	2995.2 (5489.7)	9624.7 (9362.1)	15985.4 (11034.0)
	median (IQR)	1475.3 (745.5-2957.0)	6192.0 (2536.0.5-13036.5)	13187.0 (7101.0-23062.0)

[1]Long-term exposure antigens: apical membrane antigen 1 (AMA-1), merozoite surface protein 1.19 (MSP-1.19) and glutamate-rich protein (GLURP.R2). Recent exposure (malaria infection in the past ~9 months): reticulocyte-binding protein homologue (Rh2.2030), gametocyte exported protein (GEXP18) and early transcribed membrane protein (Etramp5.Ag1). [2]Median Fluorescent Intensity is the final reading of the Luminex analysis (see 1.1. Luminex xMAP technique in Supplementary methods). SD = standard deviation. IQR = interquartile range.

Reference

1. Achan, J.; Reuling, I.J.; Yap, X.Z.; Dabira, E.; Ahmad, A.; Cox, M.; Nwakanma, D.; Tetteh, K.; Wu, L.; Bastiaens, G.J.H.; et al. Serologic Markers of Previous Malaria Exposure and Functional Antibodies Inhibiting Parasite Growth Are Associated With Parasite Kinetics Following a Plasmodium falciparum Controlled Human Infection. Clin. Infect. Dis. 2020, 70, 2544–2552. https://doi.org/10.1093/cid/ciz740.
